# Interdisciplinary Weight Loss and Lifestyle Intervention for Obstructive Sleep Apnoea in Adults: Rationale, Design and Methodology of the INTERAPNEA Study

**DOI:** 10.3390/nu11092227

**Published:** 2019-09-15

**Authors:** Almudena Carneiro-Barrera, Francisco J. Amaro-Gahete, Amparo Díaz-Román, Alejandro Guillén-Riquelme, Lucas Jurado-Fasoli, Germán Sáez-Roca, Carlos Martín-Carrasco, Jonatan R. Ruiz, Gualberto Buela-Casal

**Affiliations:** 1 Sleep and Health Promotion Laboratory, Mind, Brain and Behaviour Research Centre (CIMCYC), University of Granada, 18011 Granada, Spain; adiazroman@ugr.es (A.D.-R.); agr@ugr.es (A.G.-R.); gbuela@ugr.es (G.B.-C.); 2 EFFECTS-262 Research group, Department of Medical Physiology, School of Medicine, University of Granada, 18071 Granada, Spain; amarof@ugr.es (F.J.A.-G.); juradofasoli@ugr.es (L.J.-F.); 3 PROmoting FITness and Health through physical activity research group (PROFITH), Sport and Health University Research Institute (iMUDS), Department of Physical Education and Sports, Faculty of Sport Sciences, University of Granada, 18071 Granada, Spain; ruizj@ugr.es; 4 Unidad de Trastornos Respiratorios del Sueño, Servicio de Neumología, “Virgen de las Nieves” University Hospital, 18014 Granada, Spain; gsaezroca@gmail.com (G.S.-R.); cmartincarrasco@gmail.com (C.M.-C.)

**Keywords:** obstructive sleep apnoea, apnoea-hypopnoea index, obesity, weight loss, interdisciplinary lifestyle intervention, nutrition, exercise, sleep hygiene, smoking, alcohol

## Abstract

Obesity is a major risk factor for obstructive sleep apnoea (OSA), the most common sleep-disordered breathing related to neurocognitive and metabolic syndromes, type II diabetes, and cardiovascular diseases. Although strongly recommended for this condition, there are no studies on the effectiveness of an interdisciplinary weight loss and lifestyle intervention including nutrition, exercise, sleep hygiene, and smoking and alcohol cessation. INTERAPNEA is a randomised controlled trial with a two-arm parallel design aimed at determining the effects of an interdisciplinary tailored weight loss and lifestyle intervention on OSA outcomes. The study will include 84 males aged 18–65 with a body mass index of ≥25 kg/m^2^ and severe to moderate OSA randomly assigned to usual care (i.e., continuous positive airway pressure), or interdisciplinary weight loss and lifestyle intervention combined with usual care. Outcomes will be measured at baseline, intervention end-point, and six-month post-intervention, including apnoea-hypopnoea index (primary outcome), other neurophysical and cardiorespiratory polysomnographic outcomes, sleep quality, daily functioning and mood, body weight and composition, physical fitness, blood biomarkers, health-related quality of life, and cost-effectiveness. INTERAPNEA may serve to establish a cost-effective treatment not only for the improvement of OSA and its vast and severe comorbidities, but also for a potential remission of this condition.

## 1. Introduction

Obstructive sleep apnoea (OSA), produced by repeated upper airway collapse during sleep, has increasingly become the focus of numerous current interdisciplinary research attributed not only to its high prevalence, but also to the wide range of adverse health consequences of this condition [1]. The repeated events of complete (apnoea) or partial (hypopnoea) pharyngeal obstruction that occur while sleeping lead to intermittent hypoxic episodes, hypercapnia, sleep fragmentation, and upsurges of sympathetic activity [2]. Driven by these short-term consequences, OSA is closely related to increased morbidity and mortality [3], including cardio-metabolic disorders [4], neurocognitive abnormalities [5], impaired daily functioning and mood [6], and greater risk of vehicle and occupational accidents [7,8]. 

It has recently been estimated that up to 38% of adults suffer from OSA, being more prevalent in males, the elderly, and in those who are obese [9]. Therefore, OSA risk factors include obesity, sex, age, and adverse lifestyle habits such as sedentariness, poor nutrition, smoking, and alcohol intake [10]. According to epidemiological studies, nearly 60% of moderate to severe OSA is attributable to obesity [11], which contributes to alterations of the airway anatomy and collapsibility, respiratory modulation, resting lung volume, and neurohormonal mediators on ventilation [12]. Given the exponential increase of obesity prevalence in the overall population, which has nearly tripled since 1975—in 2016 39% of adults aged 18 years and over were obese—OSA prevalence is not only worryingly high but also likely to rise in the upcoming years [13].

The current treatment of choice is continuous positive airway pressure (CPAP) [14], a mechanical device used to maintain upper airway patency, thereby improving the main symptoms and consequences of OSA through the reduction of the number of apnoea-hypopnoea episodes per hour of sleep (i.e., apnoea-hypopnoea index, AHI) [15,16,17]. However, CPAP is a chronic day-to-day treatment—it does not cure OSA in the long-term—and its use may be rejected or abandoned due to discomfort and/or other inconveniences [18]. Most importantly, CPAP does not address the major high-risk factors of OSA, i.e., obesity and adverse lifestyles.

Hence, alternative or combined behavioural interventions including weight loss through dietary approaches and exercise, sleep hygiene, and avoidance of alcohol and tobacco consumption are required and strongly recommended in the most recent practical guidelines from the American Academy of Sleep Medicine (AASM) [14,19]. According to our recently published systematic review and meta-analysis on the effectiveness of these interventions [1], the combination of diet and exercise may be an effective treatment in improving OSA outcomes in middle-aged males with moderate to severe OSA.

Yet, the number of reported randomised controlled trials addressing both diet and exercise components as a combination was significantly low and only included effects on specific OSA outcomes such as AHI, oxygen desaturation index, and excessive daytime sleepiness [1]. Furthermore, no original studies actively focusing on the cessation of tobacco and alcohol consumption were found [1], factors which have been shown to be common in patients with OSA and associated with the worsening of this condition [20,21]. Thus, the actual effectiveness of potential interdisciplinary interventions for the improvement of the main symptoms and consequences of OSA still remains unclear. Considering the vast and severe OSA consequences and comorbidities, with obesity being a major risk factor for this condition, there is a need for well-designed studies comprising all of these aspects and evaluating the potential clinical and economic relevance of these interventions for OSA and related diseases. 

The INTERAPNEA randomised controlled trial (RCT) is aimed at implementing and testing the effectiveness of an eight-week interdisciplinary weight loss and lifestyle intervention on overweight-obese adults with CPAP-treated moderate to severe OSA. This intervention will include nutritional behaviour change, supervised-exercise, sleep hygiene, and active alcohol and tobacco cessation components, comparing the impacts on primary and secondary OSA outcomes to the standard care, i.e., CPAP therapy. Furthermore, INTERAPNEA not only pursues to analyse the effect of this intervention on OSA outcomes but also on the overall physical and psychological health of patients with moderate to severe OSA.

## 2. Methods

### 2.1. Study Design

The INTERAPNEA study (ClinicalTrials.gov ID: NCT03851653) is an RCT with a two-arm parallel design where participants will be randomly allocated to a usual care/control group (i.e., CPAP therapy) or an 8 week interdisciplinary weight loss and lifestyle intervention combined with CPAP. The study conforms to the last revised Ethical Principles for Medical Research Involving Human Subjects comprised in the Declaration of Helsinki, and approval of the study protocol was obtained from the Clinical Research Ethics Committee of the “Junta de Andalucía” (0770-N-19). All participants will receive accurate information on the study assessments and intervention, and written informed consent from each participant will be obtained prior to any data collection.

### 2.2. Study Organisation and Coordination Centre 

Adults diagnosed with moderate to severe OSA potentially meeting the inclusion criteria will be recruited from the “Virgen de las Nieves” University Hospital (Granada, Spain). Data collection at baseline and follow-ups, as well as implementation of the intervention, will be performed in two different settings of the University of Granada (Granada, Spain): Sleep and Health Promotion Laboratory of the Mind, Brain, and Behaviour Research Centre (CIMCYC), and Sport and Health University Research Institute (iMUDS). The Sleep and Health Promotion Laboratory is the coordinating centre for the study, responsible for the study design and organisation, patient recruitment process, data collection and management, randomisation and participant allocation, trial monitoring, and reporting of the study process and results.

### 2.3. Participants and Selection Criteria

Eligible participants will be adults previously diagnosed with moderate to severe OSA (AHI equal or greater than 15 [22]) from the province of Granada (Spain). They must be between 18 and 65 years old, and have a body mass index (BMI) equal to or greater than 25 kg/m^2^. A full list of the study’s inclusion and exclusion criteria are shown in Table 1. Due to the well-evidenced higher incidence and prevalence of OSA in males [9], and the differences in OSA phenotypes between men and women [23], we decided to include only male participants in the study. Furthermore, the effectiveness of non-pharmacological and non-surgical weight loss interventions have been shown to be less effective in women [1,24], such that different approaches are needed in this population with OSA.

Potential participants will be medically examined and must complete a health history revision prior to their inclusion in the study in order to ensure no hindrance/harm related to the assessment and intervention protocols. Should any incident or medical problem arise during the intervention, participants will be physically and psychologically examined and, if necessary, excluded from the study. A clinical trial liability insurance will be contracted for the INTERAPNEA study, providing legal and financial protection to the sponsor-investigators, and compensation to participants in the case of an injury or any damage incurred in and as a result of the study.

### 2.4. Recruitment and Randomisation 

#### 2.4.1. Sample Size

The sample size calculation and power of the study are based on the data of previously reported studies contrasted, combined, and synthesised in our recent systematic review and meta-analysis [1]. We considered following the formula $n=\;\frac{2{\left(Z_{\alpha }\;+\;Z_{1-\beta }\right)}^{2}\sigma^{2}}{\Delta^{2}}$, where *n* is the required sample size, *Z_α_* and *Z*_(1 − *β*)_ are the constants set by convention according to the accepted *α* error and power of the study, respectively, *σ* is the estimated standard deviation, and Δ is the expected effect size. Therefore, we expect to detect an effect size of −8.36 on AHI (pooled raw mean difference of previous trials) [1], considering a type 1 error/α error of 5% (*Z_α_* = 1.64), and a statistical power of 90% (*Z*_(1 − *β)*_ = 1.28). Regarding the estimated AHI variability, we established an *σ* of 11.98, considering the AHI pooled standard deviation of all independent samples included in our previous research [1]. As a result, the expected sample size is ≈35 participants per arm of our controlled clinical trial. However, assuming a maximum of a 17.25% drop-out rate (based on the average drop-out rate of previous studies [1]), we decided to recruit a total sample size of ≈42 participants for each study group. Thus, a total of ≈84 patients with moderate to severe OSA will be enrolled in the INTERAPNEA study. For practical and feasibility reasons, and based on our previous experience [25,26], the study will be conducted in sets of a maximum of 30 persons.

#### 2.4.2. Source of Participants

**Figure 1:**
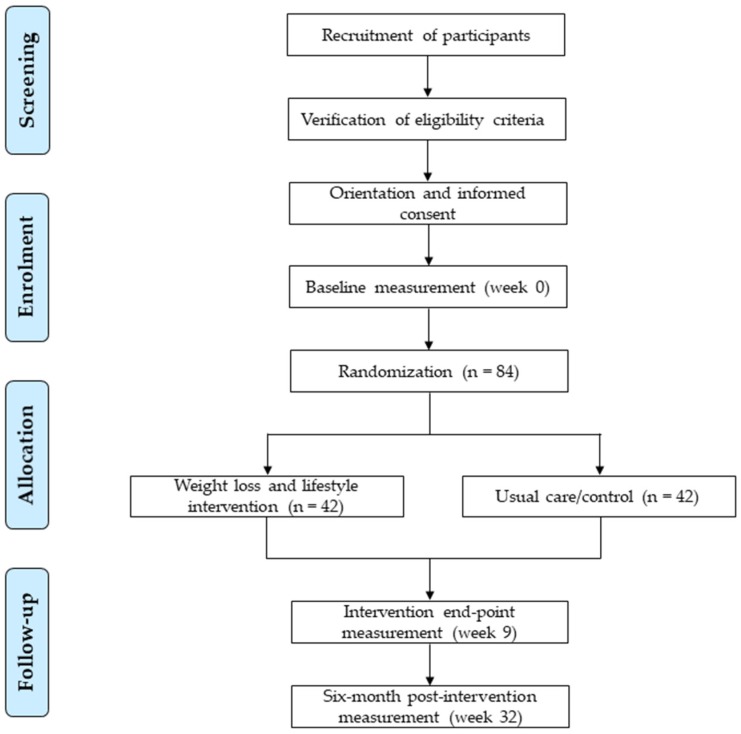
Flow diagram of the INTERAPNEA study participants.

The recruitment of participants will be performed using different strategies including enrolment from the collaborating hospital sleep unit, and use of mass media (e.g., press, magazines, radio and television news, and websites). A brief in-person or phone screening will be conducted on potentially interested participants to provide general information about the study and determine suitability of inclusion. Patients willing to participate and appearing to meet the inclusion criteria will be required to attend an in-person briefing on the rationale and study aims, inclusion and exclusion criteria, assessments to be performed, and components and characteristics of the intervention. After clarification by the research staff of any participant’s doubts or questions, signatures of informed consent will be obtained from participants that meet the eligibility criteria, and appointments for the baseline assessment will be given. Participant flow from recruitment to randomisation stages are shown in Figure 1.

#### 2.4.3. Enrolment

Upon obtaining signed informed consents, participant demographics and medical history will be collected, and a medical/physical examination will be performed to ensure feasibility of participant inclusion in the study. Subsequently, a sleep study through a complete full-night polysomnography and other sleep measurements (daytime sleepiness, sleep quality, circadian preference, functional outcomes of OSA) will be conducted on and taken from each participant. Furthermore, lifestyle habits such as diet, exercise, and tobacco and alcohol consumption will also be measured, as well as subjective health-related quality of life, and depressive and anxiety symptoms related to OSA. After completion of participant’s medical and sleep studies, objective measurements of cardiorespiratory fitness and body composition will be taken from each participant. All test trials will be scheduled over three different days during a one to two-week period.

#### 2.4.4. Randomisation and Blinding

After completing baseline measurements, eligible participants will be randomly assigned to either a control group or an interdisciplinary intervention group using computer generated simple (unrestricted) randomisation [27]. Each participant will be specifically informed of which arm they have been assigned to and requested not to reveal their allocation to the research staff involved in further assessments. Bias related to unblinded participants, treatment counsellors and/or outcome assessors affecting data validity will be addressed by achieving different levels of blinding across the study personnel and participants, where feasible. Therefore, study personnel responsible for data collection and analysis will be blinded to allocation assignments at the follow-ups, and blinding of participants to details of study manuals and hypothesis will be attained. When blinding is not possible, rigorous procedures of standardisation of data collection and intervention, through study manuals and continuous assessment of fidelity, will be followed to avoid potential bias and ensure internal and external validity of the study [28].

### 2.5. Assessment/Outcome Variables

The primary outcome of the INTERAPNEA study is the reduction in the number of apnoea and/or hypopnoea episodes per hour, i.e., AHI, assessed using a full-night ambulatory polysomnography. The main secondary outcomes include other neurophysical and cardiorespiratory polysomnographic outcomes, body weight and composition, physical fitness/cardiorespiratory fitness, and health blood biomarkers. Other variables of interest are subjective measurements of depressive and anxiety symptomatology related to OSA, impaired sleep (i.e., daytime sleepiness, sleep quality, and functional outcomes of OSA), health-related quality of life, and other lifestyle habit measurements (i.e., diet, physical activity, alcohol and tobacco consumption). All outcomes will be measured at baseline (week 0), intervention end-point (week 9), and 6 months post-intervention (week 32).

Assessment of primary and secondary outcomes will be organised and completed over three different days during a one to two-week period:

- Day 1: Potential participants will attend a medical examination and a fasting blood test at the Sleep Unit of “Virgen de las Nieves” University Hospital.
- Day 2: Eligible participants will complete a full-night in-laboratory polysomnography (PSG; the gold-standard objective testing recommended by the AASM [29]), at the Sleep and Health Promotion Laboratory (CIMCYC). In order to avoid potential CPAP influence on PSG outcomes, participants will be required to withdraw from CPAP during the week prior to the PSG at baseline and follow-ups [30]. Prior to PSG, participants will also complete a set of questionnaires measuring subjective variables related to sleep, general physical and psychological health, and lifestyle habits including diet, physical exercise, and alcohol and tobacco consumption.
- Day 3: During the third and last assessment day, participants will be required to attend the iMUDS for the measurement of anthropometric parameters, body composition and cardiorespiratory fitness.

Baseline physical activity and sleep habits will also be obtained through a seven-day self-reported daily step log and sleep diary. See Table 2 for study outcomes and measurements.

#### 2.5.1. Primary Outcome: Apnoea-Hypopnoea Index

The primary outcome of the INTERAPNEA study is AHI, defined as the number of apnoea (90% or greater drop in airflow for 10 s or longer) and hypopnoeas (30% or greater drop in airflow for 10 s or longer associated with ≥3% oxygen desaturation or an arousal) episodes per hour of sleep [31].

We will measure this outcome and other neurophysical and cardiorespiratory secondary outcomes through an in-laboratory PSG using SOMNOScreen™ PSG-Tele (SOMNOmedics, GmbH, Randersacker, Germany), or Somté PSG v2 system (Compumedics Limited, Abbotsford, Australia). The recordings will include all recommended physiologic signals such as electroencephalogram (three channels: F4-M1, C4-M1, O2-M1), electrooculogram (two channels: E1 and E2), electromyogram (two channels: submental and anterior tibialis muscles), and electrocardiogram (two channels). Cardiorespiratory measurements will include oral and nasal airflow (triple thermistor), oxyhaemoglobin saturation (SpO_2_) and pulse-rate (pulse oximeter), respiratory effort (chest and abdomen bands), and body position (sensor). All electrodes will be placed in accordance with the international 10–20 system [32], and recordings will be automatically and manually scored in 30 s epochs [33] by trained physicians using DOMINO (v2.7, SOMNOmedics, GmbH, Randersacker, Germany), or ProFusion PSG 3 (v3.3, Compumedics Limited, Abbotsford, Australia) associated computer software. All parameters, settings, filters, technical specifications, sleep stage scoring and event scoring will be performed in accordance with the AASM Manual for the Scoring of Sleep and Associated Events [31].

We will also specifically analyse AHI in rapid eye movement (REM) and non-REM sleep stages (N1, N2, and N3). Although it has been shown that REM apnoea episodes may yield more adverse cardiovascular consequences than non-REM obstructions [34], previous similar RCTs have rarely included the reduction in AHI differentiated by these sleep stages [1].

#### 2.5.2. Secondary Outcomes

##### Neurophysical and Cardiorespiratory Polysomnographic Outcomes

Secondary polysomnographic outcomes related to OSA measured by PSG, as mentioned above, are oxygen desaturation index (number of oxygen desaturation ≥3% per hour), SpO2 mean (average of oxygen saturation), SpO2 nadir (minimum oxygen saturation), arousal index (number of arousal per hour), total sleep time, sleep efficiency (total sleep time/total time in bed), sleep latency, wake after sleep onset (number of awakenings), REM sleep stage, REM latency, nonREM sleep stages (N1, N2, and N3), periodic limb movements index (PLMI; periodic limb movements per hour of sleep), and heart rate variability (HRV)/ST segment changes.

##### Physical Fitness

Cardiorespiratory fitness will be measured through a 2 km walking test, which has been widely used and validated for accurate estimation of maximum oxygen uptake (VO_2max_) [35]. Participants will be required to walk over a marked 2 km track on a firm surface wearing a heart rate monitor (Polar RS800cx, Polar Electro, Kempele, Finland). Walking time and heart rate (HR) will be recorded at the end of the test. The maximal aerobic power will then be calculated considering age, BMI, performance time, and HR with the following formula $VO_{2max}\left(\left(ml/min\right)/kg\right)=116.2-2.98*walking\;time\;\left(\sec\right)-0.11*HR-0.14*age-0.39*BMI$ [36]. Participant’s scores will be obtained and placed within a fitness category. Subjective physical fitness will also be measured using the International Fitness Scale (IFIS) [37].

##### Body Weight and Composition

Body weight and height will be measured using a calibrated scale and stadiometer (model 799, Electronic Column Scale, Hamburg, Germany) with participants wearing undergarments. Neck, chest and waist circumferences will also be measured following standard procedures recommended by the International Society for the Advancement of Kinanthropometry (ISAK) [38]. Body composition measurements including fat mass (kg), fat free mass, lean mass (kg), visceral adipose tissue (kg), and bone mineral density (g/cm^2^) will be obtained through a full-body dual energy X-ray absorptiometry (DXA) scanner (Discovery Wi, Hologic, Inc., Bedford, MA, USA). Quality controls, positioning of participants and analyses of results will be performed following the manufacturer’s recommendations. Automatic delineation of anatomic regions will be performed using APEX 4.0.2. software.

##### Blood Biomarkers

Blood samples will be obtained from participants’ antecubital vein in a supine position during the morning in a fasting state. Blood parameters will include insulin, glucose, triglycerides, total cholesterol, high-density lipoprotein cholesterol (HDL-C), low-density lipoprotein cholesterol (LDL-C), alanine transaminase (ALT), and γ-glutamyl transferase (γ-GT). Glucose levels will be measured by spectrophotometric techniques (AU5800, Beckman Coulter, Brea, California, USA). Insulin will be assessed by chemiluminescence immunoassay with paramagnetic particles (UniCel DxI 800, Beckman Coulter, Brea, California, USA). Triglycerides, total cholesterol, and HDL-C will be automatically evaluated by spectrophotometric techniques (AU5800, Beckman Coulter, Brea, California, USA). LDL-C will be considered as (Total cholesterol) − (HDL−C) − 0.45 ∗ (Triglycerides). ALT and γ-GT will be calculated by absorption spectrophotometric techniques (Beckman Coulter, Brea, California, USA). Insulin glucose ratio (insulin/glucose), LDL-C/HDL-C ratio (LDL-C/HDL-C), and triglycerides/HDL-C ratio (triglycerides/HDL-C) will also be determined.

##### Lifestyle Habits: Dietary Habits, Physical Activity, Smoking, and Alcohol Intake

Participants’ dietary habits will be evaluated using the validated 14-item Mediterranean diet screener (MEDAS), which evaluates food consumption frequency (12 items) and characteristic dietary habits of the Mediterranean diet (2 items) [39]. MEDAS items are scored with 0 or 1, the total score ranging from 0 to 14 points. The 22-item Food Behaviour Checklist (FBC) will also be used to assess participants’ food intake and habits [40]. FBC comprises seven subscales including consumption of fruit and vegetables (9 items), diet quality (4 items), fast food (3 items), dairy/calcium (2 items), sweetened beverages (2 items), meat (1 item) and food security (1 item). This instrument has been shown to be effective at evaluating dietary behaviour changes after nutrition education interventions promoting healthy diets [40].

Physical activity will be measured using daily step logs recorded by participants with a spring-levered pedometer (OcioDual, Alicante, Spain). Participants will be required to wear the pedometer all day and register the number of steps achieved per day in a seven-day step log. The average steps per day will then be calculated at baseline and follow-ups.

Regarding the remaining lifestyle habits, smoking and alcohol intake will be measured at baseline and follow-ups using seven-day self-reported tobacco and alcohol consumption logs. Recordings will include number of cigarettes/alcoholic units consumed per day, cigarette brand/type of alcoholic drink, time, situation, and perceived pleasure (from 0 to 10). The validated form of the Fagerström Test for Nicotine Dependence (FTND) [41] will also be used to assess participants’ nicotine dependence in all assessments.

##### Daily Functioning and Mood

OSA impact on daily functioning and mood will be measured through validated versions of the Functional Outcomes of Sleep Questionnaire (FOSQ) [42], Beck Depression Inventory-Fast Screen (BDI-FS) [43], State-Trait Anxiety Inventory (STAI) [44] and *Inventario de Depresión Estado-Rasgo* (IDER) [45]. It has been shown that impaired daytime functioning and depressive and anxiety symptoms are very common in patients with OSA, being higher in patients with more severe OSA and a greater BMI [46]. Hence, participants will complete a set of questionnaires on these symptoms not only to measure inclusion/exclusion criteria but also to analyse potential changes in daily functioning and mood driven by the INTERAPNEA study intervention.

##### Daytime Sleepiness, Sleep Quality and Health-Related Quality of Life

The Epworth Sleepiness Scale (ESS) [47], an 8-item Likert-based scale, will be used to obtain subjective measurements of participant’s daytime sleepiness. Excessive daytime sleepiness is the most common consequence of OSA due to sleep fragmentation and deprivation, and one of the mediating factors for other OSA outcomes such as daily functioning, social and occupational disturbances [6,7,8]. We will also include the Psychomotor Vigilance Test (PVT) [48], regarded as a potential and reliable objective measure of sleepiness, using PC-PVT v 2.0 software (Biotechnology HPC Software Applications Institute [BHSAI], https://pcpvt.bhsai.org/pcpvt/register.xhtml, Frederick, Maryland, USA) on a personal computer [49,50]. This 10 min sustained attention task consists of responding to visual stimulus randomly presented in a black screen each 2 to 10 s, to which participants have to respond by clicking the mouse, with the reaction time being registered and analysed in terms of response speed and number of lapses (reaction time >500 ms).

As sleep quality is also closely related to daily functioning, mood and, thus, participant’s general quality of life and well-being [51,52], we will measure potential benefits of the INTERAPNEA study intervention on these variables through the validated versions of the Pittsburgh Sleep Quality Index (PSQI) [53], Sleep Apnea Quality of Life (SAQLI) [54], Short-Form 36 Health Survey (SF-36) [55], and General Health Questionnaire (GHQ-28) [56].

In addition, we will subjectively measure circadian preference or individual’s chronotype using the validated reduced 5-item version of the Morningness-Eveningness Questionnaire (MEQ) [57], an outcome which has been closely related to age, BMI and, in turn, OSA [58]. Evidence suggests that evening-type chronotype may be highly associated with greater unhealthy eating behaviours, sleep disruption, poor sleep quality and mood disturbances, all playing a part in the development and severity of OSA [58,59].

##### Cost-Effectiveness Analysis Outcome

Cost-effectiveness analysis (CEA) is an essential outcome measurement to be included in RCTs in order to evaluate not only whether a new/alternative intervention produces higher beneficial effects than standard care, but also if those benefits are sufficiently significant to justify the additional costs [60,61,62]. INTERAPNEA will include CEA as an important variable considering the incremental cost-effectiveness ratio (ICER) of the interdisciplinary weight loss and lifestyle intervention related to the usual care (i.e., CPAP). Thus, following standard measures used in medical cost-effectiveness studies and international recommendations [60,61], we will calculate the ratio of the incremental costs and the incremental clinical benefits as the additional expenditure required to generate an additional unit of benefit, expressed as cost per quality-adjusted life-year (QALY) added, and calculated as $CE=\frac{Cost_{2}-Cost_{1}}{QALY_{2}-QALY_{1}}$ [62]. With regard to the cost measurements, we will follow the WHO recommendations for estimating costs contemplated in its CEA guidelines such as the cost of providing the intervention (e.g., labour, capital, and consumables), and costs of accessing the intervention (e.g., resources used and time costs related to seeking or obtaining the intervention) [60]. Furthermore, we will conduct additional sensitivity analyses considering age-weighting as well as weighting of other potential variables such as OSA severity and other secondary outcomes. Apart from calculating the cost-effectiveness ratio of the intervention related to usual care, we will consider the acceptable Spanish cost-effectiveness ratio threshold of 25.000 €–30.000 € per QALY added [63,64]. Subject to the interdisciplinary weight loss and lifestyle intervention resulting in significantly better clinical outcomes and considerably lower costs, the INTERAPNEA intervention could potentially be labelled as a ‘dominant strategy’ [62].

### 2.6. The Intervention Rationale

The design, implementation and evaluation of the INTERAPNEA study intervention components and characteristics are based on results of previous epidemiological and clinical research [1,10] as well as on international evidenced-based clinical practice guidelines for the management of OSA [14,19]. Considering our previous research [1] and with the final aim of the intervention being adaptable to actual primary health-care settings, the intervention will last eight weeks, and will be composed of five different modules: (i) Nutritional behaviour change, (ii) moderate aerobic exercise, (iii) smoking reduction and cessation, (iv) alcohol intake avoidance, and (v) sleep hygiene (see Table 3). Each component will include group-based weekly sessions of 60–90 min lead and supervised by a trained professional in the field (i.e., human nutrition and dietetics, physical activity and sport sciences, and psychology).

The key-factor of this interdisciplinary intervention will be the use of the Transtheoretical Model of Health Behaviour Change (TM) by Prochaska and Diclemente [65]. This well-evidenced model of behaviour change is based on integrating different intervention theories into an interventional approach that considers different stages, processes and principles of change with the premise of establishing sustainable health-related behaviours or habits [65]. Consciousness raising, self-reevaluation, counterconditioning, stimulus control, contingency management, goal-setting, self-monitoring, self-efficacy, and decisional balance are some of the processes and principles of change addressed by this theory and, therefore, included in the five different INTERAPNEA intervention components. Physical and dietary interventions for weight loss using strategies of TM and, thus, psychological support, have been shown to be more effective than other approaches in overweight and obese patients [66] and, specifically, in those with OSA [1].

#### 2.6.1. Nutritional Behaviour Change

Diet quality and dietary patterns have been shown to be closely related to biologic pathways involved in chronic disease etiology [67] and, specifically, to sleep disruption, fragmentation and poor sleep quality found in OSA [68]. Recent studies have shown that high-fat intake is associated with lower sleep efficiency and REM sleep and higher arousal index, whereas high-carbohydrate intake may improve sleep duration and architecture by producing reductions in sleep-onset latency and higher proportions of REM sleep [68]. Regarding intake of micronutrients, vitamin D—which has been associated with insulin resistance in OSA [69]—and magnesium deficiencies have also been related to shorter sleep duration, poorer sleep quality and higher daytime sleepiness [70,71]. Therefore, dietary components including milk, fish, fruit and vegetables may yield beneficial effects on sleep and, in turn, OSA [68].

Furthermore, evidence suggests that sleep disturbances occurring in OSA, in turn, have adverse consequences on calorie intake and expenditure, therefore, exposing a two-way relationship between dietary habits and sleep [72]. Empirical studies have revealed that sleep fragmentation and deprivation are related to higher energy intake of unhealthy foods due to increased hunger, food craving, food reward and portion size selection [73,74,75]. Neurocognitive impairments found in patients with OSA such as attention and episodic memory deficits [76] have also been associated with higher intake of saturated fats, loss of control over food intake, and thus uncontrolled eating [77].

Hence, the INTERAPNEA intervention includes a nutrition module comprising eight 60–90 min sessions (once a week) in a group format addressing dietary patterns using integrated techniques of nutrition education and behavioural change such as specific goal-setting, cognitive restructuring, stimulus control, progressive muscle relaxation, social skills and assertiveness, and problem solving skills. The nutrition education is based on the World Health Organisation’s (WHO) latest recommendations on food intake and healthy diet (see Table 4 for detailed topics) and each session will follow a three-part format: (i) Brief review of previous session and participant’s adherence to recommendations; (ii) development of the main nutrition education components of each session using an interactive group discussion layout; (iii) resolution of participant’s questions and/or concerns, and setting of specific goals. No specific or individualised diet will be indicated to participants.

#### 2.6.2. Physical Exercise

Physical exercise has been shown to be effective in enhancing OSA outcomes and health-related consequences [1,78,79]. Due to the close association between OSA and obesity, a significant and sustainable increase of physical activity could lead to a reduced body weight and, in turn, improvement of the upper airway structure, function, and resting lung volume [12]. Furthermore, physical exercise could also assist the balance of energy intake and expenditure [80], and improve respiratory centre modulation through a reduction of the high leptin and ghrelin hormone levels, which are abnormalities linked to excessive energy intake found in OSA patients [81]. Yet, some research found that exercise benefits on OSA were independent to weight loss [79], suggesting that there are other related mechanisms potentially leading to OSA enhancement such as the increase of sleep efficiency and slow wave sleep [82], and a decrease of fluid accumulation implicated in the upper airway collapse [83], both due to the direct association between physical exercise and sleep.

Therefore, the INTERAPNEA study will include an eight-week physical exercise programme consisting of weekly 60 min sessions of supervised moderated aerobic exercise (i.e., 55–65% of the heart rate reserve) and individualised goal-setting to increase daily steps per week. Previous studies have emphasised that walking may be the exercise modality to achieve higher levels of weight loss and increased cardiorespiratory fitness in adults with obesity and CPAP-treated OSA [84]. Thus, in the weekly supervised training sessions, participants will be required to walk at a moderate intensity for 60 min wearing a heart rate monitor in order to train themselves to walk at that intensity during the week. With respect to goal-setting, they will be advised to increase their daily steps by 15% per week, based on their daily steps logs.

#### 2.6.3. Sleep Hygiene

Sleep hygiene refers to the practice of certain behaviours that facilitate sleep onset and maintenance (e.g., regular sleep schedule, appropriate sleep environment, exercise-training, and healthy nutrition), and avoidance of habits interfering with sleep (e.g., daytime napping, smoking, alcohol intake, and use of hypnotics) [85]. Patients with OSA frequently exhibit poor sleep hygiene including voluntary sleep restriction, irregular sleep schedule, inappropriate sleep environment, and excessive consumption of alcohol, nicotine and/or caffeine [86]. Accordingly, previous studies have supported the inclusion of this component in the treatment of OSA as effective in improving sleep quantity and efficiency, and therefore daytime sleepiness [1,87,88].

The INTERAPNEA study intervention will include a sleep hygiene module comprising 60 min sessions supervised by a psychologist, specialised in the evaluation and treatment of sleep disorders. As most sleep hygiene topics will be covered in simultaneous modules, there will be four sessions distributed over the eight weeks of the intervention, consisting of sleep hygiene education on causes of sleep disturbances and mistaken sleep related knowledge (see Table 5). Sessions will also be based on treating those frequent inadequate sleep habits found in patients with OSA, i.e., sleep restriction, irregular schedule and inappropriate sleep environment.

#### 2.6.4. Reduction and Avoidance of Tobacco Consumption

Smoking has been related to the worsening of OSA via different mechanisms such as changes in sleep architecture and increase of arousal threshold from sleep, reduction of the upper airway muscle tones and neural reflexes, and increased inflammation of the upper airway, all due to nicotine and smoke inhalation [21]. In turn, OSA could also be a predisposing factor for smoking addiction, with nicotine acting as a reward or self-medication for the depressive and anxiety symptoms commonly found in OSA [89]. Although this association has been well elucidated, there is no empirical evidence of the potential beneficial effects of smoking cessation on OSA as, surpringsily, there are no studies focusing on active smoking cessation interventions in patients with this condition [1].

Therefore, we will include a smoking reduction and avoidance module in the INTERAPNEA study intervention. Participants with smoking addiction who are willing to quit will be required to attend a weekly 60–90 min session over eight weeks lead by two clinical psychologists. The specific intervention is based on the group behaviour therapy for smoking cessation by Becoña et al. [90]. This therapy seeks the progressive reduction of tobacco consumption through the use of nicotine and cigarette fading [91], as well as behaviour-change techniques such as information on smoking, self-monitoring, stimulus control, avoidance of withdrawal symptoms, and relapse prevention (see Table 6). Nicotine and cigarette fading has been shown to be the most effective method to reduce and stop smoking with abstinence rates of 86% at the end of treatment and nearly 60% at a 12 month follow-up [92].

Thus, participants will be mainly required to keep a daily record of the number of cigarettes smoked, and triggers for smoking (self-monitoring), change the type of cigarette smoked to a lesser nicotine content brand each week (30%, 60% and 90% nicotine reductions from baseline), reduce the number of cigarettes smoked by 30% weekly, and avoid smoking in three different situations per week (stimulus control). Through the sessions, other behaviour change techniques will be implemented such as discussions on the health consequences of smoking and quitting (motivation), muscle and cognitive relaxation techniques to address withdrawal symptoms, and identification of high-risk situations for smoking and problem-solving skills (relapse prevention).

#### 2.6.5. Reduction and Avoidance of Alcohol Intake

Alcohol intake has also been related to the development and worsening of OSA not only for its direct and indirect effects on weight gain but also due to its negative impact on breathing parameters during sleep [20]. Recent meta-analyses on alcohol and risk of sleep apnoea emphasised that alcohol intake increases the risk of breathing cessation episodes by 25%, thus increasing AHI and reducing mean SaO_2_ during sleep [93]. Potential explanations for these adverse consequences may be the alcohol-related hypotonia of oropharyngeal muscles during sleep, and depression of the arousal response to asphyxia, both caused by the alcohol depressant effects on the central nervous system [94].

Therefore, the INTERAPNEA study intervention will include an alcohol intake reduction and avoidance module supervised by two clinical psychologists. As we will be treating excessive alcohol intake as opposed to alcohol dependence, this module will last eight weeks comprising fortnightly sessions of 60 min. Similar to the smoking cessation module, the main content of this specific component is the progressive reduction of alcohol intake in those participants with no alcohol addiction but excessive consumption (see Table 7). Thus, participants will be indicated to reduce the number of units of alcohol consumed per day/week by 30% each week, keeping a log of alcohol-consumption per day including units of alcohol consumed and triggers of consumption. During the sessions, participants will receive detailed information of alcohol general and specific to OSA health-related consequences. Furthermore, behaviour change techniques such as stimulus control, muscle and cognitive relaxation and problem-solving skills related to alcohol consumption will be used.

### 2.7. Standard Care/Control Group

Participants with moderate-severe OSA randomly assigned to the usual care group (control) will receive, apart from CPAP treatment, general advice on weight loss and lifestyle changes from a sleep disordered-breathing specialist. Informative leaflets describing the positive effects of healthy nutrition, physical activity, sleep hygiene and tobacco and alcohol avoidance for OSA will also be provided to these participants. Additionally, the opportunity to receive the INTERAPNEA study intervention will be offered to this control group after the six month follow-up.

### 2.8. Assessment of Compliance and Integrity of Intervention

Integrity of the intervention and treatment fidelity will be evaluated and ensured including the design and implementation of different strategies of process assessment, monitoring and enhancement in order to guarantee internal and external validity of the trial [95].

Firstly, regarding the study design and provider of intervention training, we developed a comprehensive hand-book for the qualified INTERAPNEA study intervention providers/professionals/training personnel of each module (nutrition, physical exercise, sleep hygiene, and tobacco and alcohol consumption). Each intervention manual identifies the theoretical model of the intervention and provides detailed descriptions of session objectives, treatment guidelines in accordance with each objective (i.e., contents, tasks and activities, recommendations, and timing), participant’s homework, and material needed for each session. We will also provide each participant with an adapted patient-handbook for each intervention component including descriptions of sessions, and work and logging sheets.

Secondly, we will ensure fidelity in the treatment delivery, receipt, and enactment through the use of these intervention protocols/manuals and monitoring of the implementation. Regarding the treatment delivery, the standardisation of the intervention will support the protocol adherence of providers and the treatment differentiation (i.e., the delivery of the target treatment and no other). Furthermore, we will include a check-list for provider’s self-report concerning the achievement of session objectives. With respect to the treatment receipt and enactment, fidelity will be assessed and confirmed through different strategies such as the structuring of the intervention around achievement-based objectives, collecting and reviewing of participants self-monitored data (daily steps log, sleep diaries, alcohol and tobacco consumption records), and information delivery in different formats (e.g., written in the handbooks, and verbal and visual in the sessions).

Finally, apart from the above mention strategies, we will consider complementary approaches in order to reduce participant drop-out rates and increase adherence such as prevention of commitments or vacation periods, use of well-equipped and conditioned facilities, and supervision by a qualified and certified pair of providers in each session, motivating and supporting participants. Participants’ attendance to each intervention session will be recorded by providers, and phone-calls will be made to assess causes of absence and determine further participation in the intervention.

### 2.9. Analytical Approach and Statistical Power/Data Management

We will perform descriptive and exploratory preliminary analyses of all the study variables to reveal violations of statistical assumptions, distributions, imbalances between the study groups, associations between study variables, covariates/confounders, amount of missing data, and drop-out patterns. Covariates or confounders will be secondarily included in the main statistical analysis, evaluating therefore, their impact on the results. To ensure that conclusions are robust, attrition bias will be overcome through intention-to-treat-analysis (ITT), including all participants as originally allocated after randomisation using multiple imputation methods for predicting end-points missing values. Per-protocol comparison of groups, including only those participants who fully completed the originally allocated treatment, will also be computed and compared with ITT analysis results.

The intervention effects on primary and secondary study outcomes will be assessed through multi-level mixed analyses using the package *nlme* [96] from the R statistical program. This method will allow us to analyse differences including time and group allocation as within and between subject factors, respectively, adjusting for potential covariates and levels such as group or set of participants. Therefore, group/condition will be considered as a fixed variable (intervention group compared to control group), and sets/waves of participants as a random factor. With these specifications, we will be able to control the intra-class correlation (per set of participants nested within conditions) between scores at baseline and post-test or follow-up. We will adjust the model including the baseline values of outcomes that are to be analysed in the specific analysis as covariates, as well as other potential covariates such as age, OSA severity, BMI, motivation to change, and attrition propensity. The latter will be calculated using a model predicting the actual attrition with baseline values [97]. Baseline measurements included in these attrition propensity prediction models will include set of participants, participant’s allocation, age, OSA severity, BMI, and motivation to change. The primary and secondary study outcomes that are to be analysed will be those previously mentioned in the outcomes section.

Lastly, we will also estimate standardised effect sizes using Cohen’s d coefficients as the mean difference between the mean change in intervention and control groups from baseline to post-intervention divided by the mean baseline standard deviation [98]: *d* = [((${\bar{X}}_{pre,E}-{\bar{X}}_{pos,E}$) − (${\bar{X}}_{pre,C}-{\bar{X}}_{pos,C}$))/${\bar{S}}_{pre}$].

## 3. Potential Impact of INTERAPNEA

OSA is a global health issue with a concerning and increasing prevalence associated with the rising epidemic of obesity [9,13]. Both these related conditions are predisposing factors for the development and worsening of metabolic dysfunctions, type II diabetes, and, in turn, life-threating cardiovascular diseases such as heart failure, atrial fibrillation, coronary artery disease and stroke [99]. Due to these vast and severe health consequences, besides the direct cost of OSA diagnosis, treatment, and workplace and motor vehicle accidents produced by daytime sleepiness, OSA has become a substantial clinical and economic burden on the health system [100].

An epidemiological study by Hillman et al. [101], concluded that the overall cost of sleep disorders in Australia—with OSA as the most prevalent condition—was $7494 million in 2004 (population: 20.1 million), including direct health costs (i.e., sleep disorders and associated conditions), indirect financial costs (i.e., work-related injuries, motor vehicle accidents, and other production losses), and nonfinancial costs (net cost of suffering). Taking into account that the prevalence of OSA has dramatically increased in recent years (9% to 38% in the overall population) [9], the cost should respectively now be ominously higher. Other retrospective and longitudinal studies have also emphasised the major clinical and financial costs of OSA by reporting significantly greater healthcare utilisation by patients with this condition compared to those without OSA [102,103,104,105]. Furthermore, a recent study by Derose et al. [106] concluded that even after the provision of positive airway pressure, the rates of acute care and medication use of patients with OSA did not reduce over several years of follow-up.

The INTERAPNEA study is aimed at demonstrating the potential and beneficial effects of a non-pharmacological and non-surgical tailored weight loss and lifestyle intervention for the management, improvement, and even complete remission of OSA. Although a number of studies have separately shown that physical exercise and diet may improve OSA primary outcomes, there is a lack of studies including a combination of both weight loss components [1]. Furthermore, there are no studies including active intervention components addressing tobacco and/or alcohol avoidance in patients with this condition [1] despite the well-evidenced severe consequences that smoking and alcohol intake have on OSA and comorbid diseases [20,21,89,93,94].

To our knowledge, this is the first study to describe the effects of a well-established interdisciplinary weight loss and lifestyle intervention on the primary and secondary outcomes of OSA, and other important physical and psychological health-related outcomes such as blood biomarkers, body composition, cardiovascular risk, daytime functioning and mood, and general quality of life. The inclusion of all these secondary outcomes, in turn, will potentially allow us to determine which may be the key variables mediating and/or predicting the main changes in OSA. The use of objective measurements ensuring validity of results such as full-night PSG, blood test, body composition, and cardiorespiratory fitness, besides the addition of psychological/coaching support on the design and implementation of the intervention components, provides the INTERAPNEA study with unique and strong characteristics in this field of research.

In conclusion, the INTERAPNEA study will overcome all the shortcomings found in previous OSA research and, therefore, our findings will have a potential impact not only on the knowledge and management of this condition but also on high-risk comorbidities such as obesity, type II diabetes, cardiovascular disease, and neurocognitive dysfunctions. Considering the feasibility of the intervention in real life settings, it may contribute to the standardisation of a cost-effective treatment for preventing, improving and/or curing the severe health-consequences of this increasingly common sleep-disordered breathing.

## Figures and Tables

**Table 1:** Eligibility criteria.

Inclusion Criteria	Exclusion Criteria
Men aged 18–65 years CPAP-treated moderate to severe OSA (AHI equal to or greater than 15 events/h) BMI equal to or greater than 25 kg/m^2^ Not participating in a weight loss program Willing to provide informed consent and acceptance of random group assignment	Presence of any other primary sleep disorder Presence of any mental disorder (including depression, anxiety, and addiction to alcohol or other substances) Presence of any other severe organic disease, except for those comorbid to OSA Regular use of neuroleptic, sedative or hypnotic drugs, or any other medication that may cause sleep disturbances or increased daytime sleepiness

AHI = apnoea-hypopnoea index; BMI = body mass index; CPAP = continuous positive airway pressure; OSA = obstructive sleep apnoea.

**Table 2:** INTERAPNEA study outcomes and measurements.

Variable	Measurement	Assessment
General health history and sociodemographic information	General medical examination (i.e., anamnesis, physical exploration, vital measurements, etc.)	Week 0
	Clinical and socio-demographic interview	Week 0
	Fasting blood test	Week 0, 9, 32
Sleep quality and health-related quality of life		
Sleep habits	Sleep diary	Week 0, 9, 32
Circadian preference/chronotype	Morningness-Eveningness Questionnaire	Week 0, 9, 32
Sleep quality	The Pittsburgh Sleep Quality Index	Week 0, 9, 32
Daytime sleepiness	Epworth Sleepiness Scale	Week 0, 9, 32
	Psychomotor Vigilance Test	Week 0, 9, 32
Perceived health-related quality of life	Sleep Apnea Quality of Life	Week 0, 9, 32
	Short-Form 36 Health Survey	Week 0, 9, 32
	General Health Questionnaire	Week 0, 9, 32
Objective sleep		
Neurophysiological outcomes	Polysomnography equipment	Week 0, 9, 32
Cardiorespiratory outcomes	Polysomnography equipment	Week 0, 9, 32
Body weight and composition		
BMI and anthropometric measurements	Weight and height measurement, and neck, chest and waist circumferences	Week 0, 9, 32
Body composition	Dual Energy X-ray Absorptiometry	Week 0, 9, 32
Lifestyle habits		
Physical exercise habits	Spring-levered pedometer and daily step logs	Week 0, 9, 32
Dietary habits	Food Behaviour Checklist	Week 0, 9, 32
	Mediterranean Diet Adherence Screener	Week 0, 9, 32
Tobacco dependence and consumption	Self-reported tobacco consumption logs	Week 0, 9, 32
	The Fagerstrom Test for Nicotine Dependence	Week 0, 9, 32
Alcohol consumption	Self-reported alcohol consumption logs	Week 0, 9, 32
Physical fitness		
Cardiorespiratory fitness	2 km walk test	Week 0, 9, 32
Subjective physical fitness	International Fitness Scale	Week 0, 9, 32
Daily functioning and mood		
Functional outcomes related to sleepiness	Functional Outcomes of Sleep Questionnaire	Week 0, 9, 32
Subthreshold anxiety symptoms	State-Trait Anxiety Inventory	Week 0, 9, 32
Subthreshold depression symptoms	Beck Depression Inventory-Fast Screen	Week 0, 9, 32
	Inventario de Depresión Estado-Rasgo	Week 0, 9, 32

**Table 3:** Description and timing of the INTERAPNEA intervention modules and components.

Module	Objectives/Description	Number of Sessions	Frequency of Sessions	General Behavioural Change Techniques
Nutritional behaviour change	Nutrition education and dietary patterns change	8	Once a week	Motivation and preparation for action Goal-setting and action-planning Self-monitoring and functional behavioural analysis Review of behavioural goals, action plans, and adherence Problem solving and social skills Self-efficacy, maintenance, and relapse prevention
Physical exercise	Supervised moderate aerobic exercise and increase daily steps by 15% each week	8	Once a week	
Sleep hygiene	Change of inappropriate sleep habits: Insufficient sleep, consumption of coffee, alcohol and tobacco, and inappropriate sleep schedule and environment	4	Once every two weeks	
Tobacco cessation	Nicotine and cigarette fading: Reduction of nicotine and number of cigarettes by 30% each week	8	Once a week	
Alcohol avoidance	Alcohol consumption fading: Reduction of alcohol consumption by 30% each week	4	Once every two weeks	

**Table 4:** Description of nutrition education per session.

Session	Nutrition Education Topics
Session 1	Adverse consequences of obesity, importance of healthy nutrition and body composition on health, and positive effects of changes in nutrition.
Session 2	Maintenance of a healthy nutrition based on the Harvard Plate model: increasing consumption of healthy food (vegetables, fruits, legumes, nuts, extra virgin olive oil, fish and shellfish, white meat, eggs and herbs) and decreasing consumption of unhealthy food (ultra-processed foods, excessive salt consumption, processed meats, red meat, alcohol, and high-calorie foods and beverages).
Session 3	Food myths and health risks of miracle diets.
Session 4	Strategies to improve satiety and decrease appetite: Decreasing dishes dietary energy density, choosing food with low dietary energy density, managing dietary fat intake, including enough fibre and protein, limiting sugar and ultra-processed foods, choosing water and low-calorie beverages, and managing portion sizes.
Session 5	Healthy breakfast and snacks: Avoiding unhealthy breakfasts and snacks and making them healthier.
Session 6	Healthy cooking, food purchase and choices when eating out.
Session 7	How to read nutritional labels of food and distinguish between healthy and unhealthy food.
Session 8	Nutritional strategies to improve sleep quality.

**Table 5:** Summary of components of the sleep hygiene module per session.

Session	Intervention Objectives/Components
Session 1	Goal-setting and action-planning: Objective specification and commitment Self-monitoring: Sleep diary Psychoeducation: What is sleep hygiene? Cognitive restructuring: Irrational, false or inaccurate beliefs about sleep Breathing and relaxation techniques: Diaphragmatic breathing and progressive muscle relaxation
Session 2	Review of behavioural goals, action plans, and compliance (participant’s homework) Self-monitoring: Sleep diary Psychoeducation: Voluntary sleep restriction; coffee, alcohol and tobacco consumption before sleep; and irregular sleep schedule and environment Cognitive restructuring: Irrational, false or inaccurate beliefs about sleep Review of diaphragmatic breathing and progressive muscle relaxation
Session 3	Review of behavioural goals, action plans, and compliance (participant’s homework) Self-monitoring: Sleep diary Vicarious and self-reinforcement: Changes in sleep habits achieved and benefits Stimulus control and bedtime restriction Review of diaphragmatic breathing and progressive muscle relaxation
Session 4	Review of behavioural goals, action plans, and compliance (participant’s homework) Self-monitoring: Sleep diary Vicarious and self-reinforcement: Changes in sleep habits achieved and benefits Review of all intervention components: Main factors of sleep hygiene, diaphragmatic breathing and progressive muscle relaxation, stimulus control and bedtime restriction Maintenance and relapse prevention: Analysis of high risk situations for unhygienic sleep.

**Table 6:** Summary of components of the smoking cessation module per session.

Session	Intervention Objectives/Components
Session 1	Goal-setting and action-planning: Objective specification and commitment Self-monitoring: Cigarette consumption logs Psychoeducation: Cigarette components and smoking consequences Cognitive restructuring: Irrational, false or inaccurate beliefs about smoking cessation Stimulus control: Reduction strategies Nicotine and cigarette fading: Reduction of nicotine/number of cigarettes by 30% from baseline Breathing and relaxation techniques: Diaphragmatic breathing and progressive muscle relaxation
Session 2	Review of behavioural goals, action plans, and compliance (participant’s homework) Self-monitoring: Cigarette consumption logs Cognitive restructuring: Irrational, false or inaccurate beliefs about smoking cessation Stimulus control: Smoking avoidance in three different situations and other reduction strategies Nicotine and cigarette fading: Reduction of nicotine/number of cigarettes by 30% from week 1 Maintenance and relapse prevention: Avoidance of withdrawal symptoms Review of diaphragmatic breathing and progressive muscle relaxation
Session 3	Review of behavioural goals, action plans, and compliance (participant’s homework) Self-monitoring: Cigarette consumption logs Vicarious and self-reinforcement: Changes in smoking achieved and benefits Stimulus control: Smoking avoidance in six different situations and other reduction strategies Nicotine and cigarette fading: Reduction of nicotine/number of cigarettes by 30% from week 2 Maintenance and relapse prevention: Avoidance of withdrawal symptoms Review of diaphragmatic breathing and progressive muscle relaxation
Session 4	Review of behavioural goals, action plans, and compliance (participant’s homework) Self-monitoring: Cigarette consumption logs Vicarious and self-reinforcement: Changes in smoking achieved and benefits Stimulus control: Smoking avoidance in nine different situations and other reduction strategies Nicotine and cigarette fading: Reduction of nicotine/number of cigarettes by 30% from week 3 Maintenance and relapse prevention: Avoidance of withdrawal symptoms Review of diaphragmatic breathing and progressive muscle relaxation
Session 5	Review of behavioural goals, action plans, and compliance (participant’s homework) Self-monitoring: Cigarette consumption logs Vicarious and self-reinforcement: Changes in smoking achieved and benefits Abstinence planning: Setting the day when abstinence starts Problem solving and social skills: High risk situations for smoking and alternative behaviours Maintenance and relapse prevention: Avoidance of withdrawal symptoms Review of diaphragmatic breathing and progressive muscle relaxation
Sessions 6, 7, 8	Review of behavioural goals, action plans, and compliance (participant’s homework) Self-monitoring: Cigarette consumption logs/review of abstinence Abstinence planning: Setting the day when abstinence starts Vicarious and self-reinforcement: Changes in smoking achieved and benefits Problem solving and social skills: High risk situations for smoking and alternative behaviours Review of diaphragmatic breathing and progressive muscle relaxation Maintenance and relapse prevention: Difference between lapse and relapse

**Table 7:** Summary of components of the alcohol avoidance module per session.

Session	Intervention Objectives/Components
Session 1	Goal-setting and action-planning: Objective specification and commitment Self-monitoring: Alcohol-consumption logs Psychoeducation: Alcohol consumption and adverse consequences for obstructive sleep apnoea Cognitive restructuring: Irrational, false or inaccurate beliefs about alcohol consumption Alcohol fading: Reduction of alcohol consumption/number of alcoholic drinks by 30% from baseline Breathing and relaxation techniques: Diaphragmatic breathing and progressive muscle relaxation
Session 2	Review of behavioural goals, action plans, and compliance (participant’s homework) Self-monitoring: Alcohol-consumption logs Cognitive restructuring: Irrational, false or inaccurate beliefs about smoking cessation Alcohol fading: Reduction of alcohol consumption/number of alcoholic drinks by 30% from week 1 Stimulus control: Reduction/abstinence strategies Review of diaphragmatic breathing and progressive muscle relaxation
Session 3	Review of behavioural goals, action plans, and compliance (participant’s homework) Self-monitoring: Alcohol-consumption logs Vicarious and self-reinforcement: Changes in alcohol consumption achieved and benefits Abstinence planning: Setting the day when abstinence starts Stimulus control: Reduction/abstinence strategies Review of diaphragmatic breathing and progressive muscle relaxation
Session 4	Review of behavioural goals, action plans, and compliance (participant’s homework) Self-monitoring: Alcohol-consumption logs Vicarious and self-reinforcement: Changes in alcohol consumption achieved and benefits Problem solving and social skills: High risk situations for drinking alcohol and alternative behaviours Review of all intervention components: Diaphragmatic breathing and progressive muscle relaxation, stimulus control Maintenance and relapse prevention: Difference between lapse and relapse
